# Validity and risk of adopting PGA ≤ 2 as a remission criteria of Boolean in clinical practice in patient with rheumatoid arthritis

**DOI:** 10.1038/s41598-022-07046-1

**Published:** 2022-02-22

**Authors:** Ichiro Yoshii, Tatsumi Chijiwa, Naoya Sawada

**Affiliations:** 1 Department of Rheumatology and Musculoskeletal Medicine, Yoshii Hospital, 6-7-5 Nakamura-Ohashidori, Shimanto City, Kochi Prefecture 787-0033 Japan; 2 Department of Rheumatology, Kochi Memorial Hospital, 4-13 Shiromi-cho, Kochi, 780-0824 Japan; 3 Department of Rheumatology, Dohgo Onsen Hospital, 21-21 Himetsuka-Otsu, Matsuyama, Ehime Prefecture 790-0858 Japan

**Keywords:** Rheumatic diseases, Rheumatoid arthritis, Biomarkers, Prognostic markers

## Abstract

Validity and risk of setting patient’s global assessment (PGA) ≤ 2 as a Boolean remission criteria substituting PGA ≤ 1 in treating rheumatoid arthritis (RA) was investigated. Patients were recruited from an area cohort, of whom attained Boolean remission (Boolean-1) or near remission with PGA ≤ 2 and the rest components were ≤ 1 (Boolean-2). Simplified disease activity index (SDAI) score was compared according to the criteria variations. A total of 517 patients were studied. Mean SDAI score of patients with Boolean-1 was significantly lower than that of patients with Boolean-2 at acquisition. The trend was evident in the patients who attained Boolean-1 remission. Mean SDAI score at acquisition, 6 months after, and 1 year after of patients who attained Boolean-2 first and then Boolean-1, was significantly inferior to that of patients who attained the remissions at the same time. The mean SDAI score at month 6 in the Boolean-2 was not SDAI remission at all. We concluded that setting PGA ≤ 2 as a remission criteria may not have statistical difference in disease activity from PGA ≤ 1, however, there was an determinant risk to misread that includes patient who losses clinical remission after acquisition.

## Introduction

Treat-to-target (T2T) strategies are now the global mainstay of treatment for rheumatoid arthritis (RA)^1^. One of the main points is to aim for early clinical remission and to set the basic disease activity index and to monitor patients^2^. ACR/EULAR Boolean remission is the most stringent measure of disease activity^3^, and it is known that patients who achieve Boolean remission, which is defined as to attain one or less in all of tenderness joint count (TJC), swollen joint count (SJC), patient's comprehensive evaluation (PGA), and C-reactive protein (CRP)^4,5^, can maintain stable disease activity control and activities of daily living for a long period after achieving remission^6^.

The reason for the difficulty in achieving a Boolean remission is the PGA score^7^. It is difficult to achieve a PGA score of ≤ 1, and it has been argued that even mildly lax criteria can be regarded as a clinical remission, which may lead to assurance of maintenance of activities of daily living (ADL)^8,9^. Recent reports have argued that even with PGA ≤ 2, a patient's ADL after 1 year of acquired remission changes only slightly compared with PGA ≤ 1, and PGA ≤ 2 may serve as a remission criterion^10^.

Of the four components of the Boolean remission criteria, only PGA is a patient-related outcome, and the assessment is patient subjective. SJC, TJC, and CRP reflect inflammatory status, and their trends are often associated^11^. However, it cannot be denied that PGA alone deviates from the three other indicators that are often encountered in clinical practice and confuses the assessment of the efficacy of RA therapy^7,12^. On the other hand, PGA correlates well with patient pain, and PGA minimization works well for pain relief^13^. The pain is an important decision index for the RA patient, and it cannot be judged that the RA treatment is good for the patient, if it is not improved. When pain is also taken into account as a clinical decision index, Boolean remission is considered to be a rational index configuration.

What are the clinical differences between PGA ≤ 1 and PGA ≤ 2? This study sought to evaluate differences in clinical practice between PGA ≤ 1 based Boolean remissions and PGA ≤ 2 based Boolean remissions in a retrospective cohort study.

## Results

A total of 517 patients consisted of 507 in Group-1 (a patient group who attained Boolean-1 remission with all of four components (TJC, SJC, CRP, and PGA) achieved ≤ 1) and 517 in Group-2 (a patient group who attained Boolean-2 remission with TJC, SJC, and CRP ≤ 1, and PGA ≤ 2), including 414 in Group-same1&2 (a patient group who fulfilled Boolean-1 and Boolean-2 remission at the same time), 93 in Group-step1to2 (a patient group who achieved Boolean-2 remission and then achieved Boolean-1 later), and 10 in Group-only2 (a patient group who did not achieved Boolean-1 remission but achieved Boolean-2 remission). 73.2% in Group-1 and 72.7% in Group-2 were female, compared with 72.2% in Group-same1&2, 77.4% in Group-step1to2, and 50.0% in Group-only2. Mean age at baseline was 65.6 and 65.7 years for Group-1 and Group-2, respectively, whereas it was 65.7, 65.1 and 70.0 years for Group-same1&2, Group-step1to2, and Group-only2, respectively. The demographic and clinical characteristics at baseline, initial visit, and during clinical course were as shown in Table 1.

**Table 1:** Demographic and clinical characteristics of groups.

		Group-1 (n = 507)	Group-same1&2 (n = 414)	Group-step1to2 (n = 93)	Group-only2 (n = 10)	Group-2 (n = 517)
	Female (%)	73.2	72.2	77.4	50.0	72.7
At first consult	Age (years)	64.3 (13.9)	64.6 (14.1)	63.5 (13.1)	70.0 (19.2)	64.5 (14.1)
	Disease duration (years)	6.2 (8.1)	6.7 (8.6)	4.3 (5.1)	6.2 (9.0)	6.2 (8.1)
	ACPA positive rate (%)	80.1	81.2	75.7	55.0	79.6
	RF positive rate (%)	77.3	77.4	76.7	65.0	77.0
	SDAI	22.0 (21.8)	21.6 (21.8)	23.6 (21.7)	19.3 (18.2)	22.0 (21.8)
	HAQ-DI	0.463 (0.578)	0.457 (0.580)	0.489 (0.568)	0.700 (0.731)	0.468 (0.582)
	PS-VAS	34.1 (29.1)	33.0 (29.8)	39.1 (25.0)	40.4 (19.3)	34.2 (28.9)
	SHS	46.9 (62.2)	48.5 (65.6)	39.6 (43.3)	57.3 (91.1)	47.1 (62.9)
	Time span from first Consultation to Boolean-1 (months)	14.9 (14.6)	13.9 (14.2)	19.1 (16.1)		14.7 (14.4)
	Time span from first Consultation to Boolean-2 (months)	13.0 (13.1)	13.9 (14.2)	9.2 (7.2)	7.8 (6.4)	12.9 (12.9)
	Time span from Boolean-1 to Boolean-2 (months)	1.9 (1.4)	0.0	9.9 (8.8)		1.9 (1.4)
	MTX administration rate by Boolean-1	71.1	69.6	77.4		71.1
	MTX administration rate by Boolean-2	70.4	69.7	74.2	70.0	70.5
	average MTX dosage by Boolean-1 (mg/week)	8.0 (2.6)	7.9 (2.6)	8.2 (2.8)		8.0 (2.6)
	average MTX dosage by Boolean-2 (mg/week)	7.9 (2.6)	7.9 (2.6)	8.1 (2.7)	7.4 (2.5)	7.9 (2.6)
	GCS administration rate by Boolean-1 (%)	35.7	35.0	38.7		35.0
	GCS administration rate by Boolean-2 (%)	35.1	35.0	35.5	30.0	35.0
	average GCS dosage by Boolean-1 (mg/day)	3.4 (2.5)	3.5 (2.4)	3.2 (2.8)		3.4 (2.5)
	average GCS dosage by Boolean-2 (mg/day)	3.5 (2.7)	3.5 (2.4)	3.6 (3.6)	5.4 (2.5)	3.6 (2.7)
	b/tsDMARD administration rate by Boolean-1	20.0	17.3	32.3		20.0
	b/tsDMARD administration rate by Boolean-2	19.3	17.3	28.0	0.0	18.9
	SDAI at Boolean-1	1.07 (1.04)	1.07 (1.07)	1.06 (0.90)		1.07 (1.07)
	SDAI at Boolean-2	1.43 (1.35)	1.07 (1.07)	3.06 (1.28)	2.97 (1.17)	1.46 (1.37)
	HAQ-DI at Boolean-1	0.416 (0.569)	0.410 (0.564)	0.440 (0.588)		0.416 (0.574)
	HAQ-DI at Boolean-2	0.415 (0.564)	0.410 (0.564)	0.435 (0.564)	0.700 (0.731)	0.421 (0.569)
	PS-VAS at Boolean-1	15.3 (20.5)	14.9 (20.3)	17.5 (21.1)		15.3 (20.5)
	PS-VAS at Boolean-2	15.5 (19.4)	14.9 (20.3)	18.6 (14.0)	27.4 (16.4)	15.8 (19.4)
	SHS at Boolean-1	46.2 (61.8)	48.3 (65.2)	39.3 (43.6)		46.2 (61.8)
	SHS at Boolean-2	46.1 (61.5)	48.3 (65.2)	39.6 (43.3)	57.3 (91.1)	46.2 (62.00)
	annual change of SHS after Boolean-1	–1.0 (1.8)	–1.0 (1.8)	–1.0 (1.8)		–1 (1.8)
	annual change of SHS after Boolean-2	–1.0 (1.8)	–1.0 (1.8)	–0.9 (2.0)	1.0 (2.3)	–0.9 (1.9)

The values are presented as mean (SD) unless indicated otherwise.

Group-1: a patient group who attained Boolean remission with all of tenderness joint count (TJC), swollen joint count (SJC), patient's global assessment (PGA), and serum C-reactive protein level (CRP) ≤ 1 (Boolean-1) during follow up ; Group-2, a patient group who attained Boolean near remission with TJC, SJC, CRP ≤ 1, and PGA ≤ 2 (Boolean-2) during follow up ; Group-same1&2, a patient group who attained Boolean-1 and Boolean-2 at the same time ; Group-step1to2, a patient group who attained Boolean-2 first and then achieved Boolean-1 ; Group-only2, a patient group who attained Boolean-2 but not attained Boolean-1.

Abbreviations: ACPA, anti-citrullinated polypeptide antibodies ; RF, rheumatoid factor ; SDAI, simplified disease activity score ; HAQ-DI, Health Assessment Questionnaire Disability Index ; PS-VAS, pain score with visual analog scale ; SHS, Sharp/van der Heijde score ; MTX, methotrexate ; GCS, glucocorticoid steroid ; b/tsDMARD, biologic or targeted synthetic disease-modifying anti-rheumatic drug.

**Figure 1:**
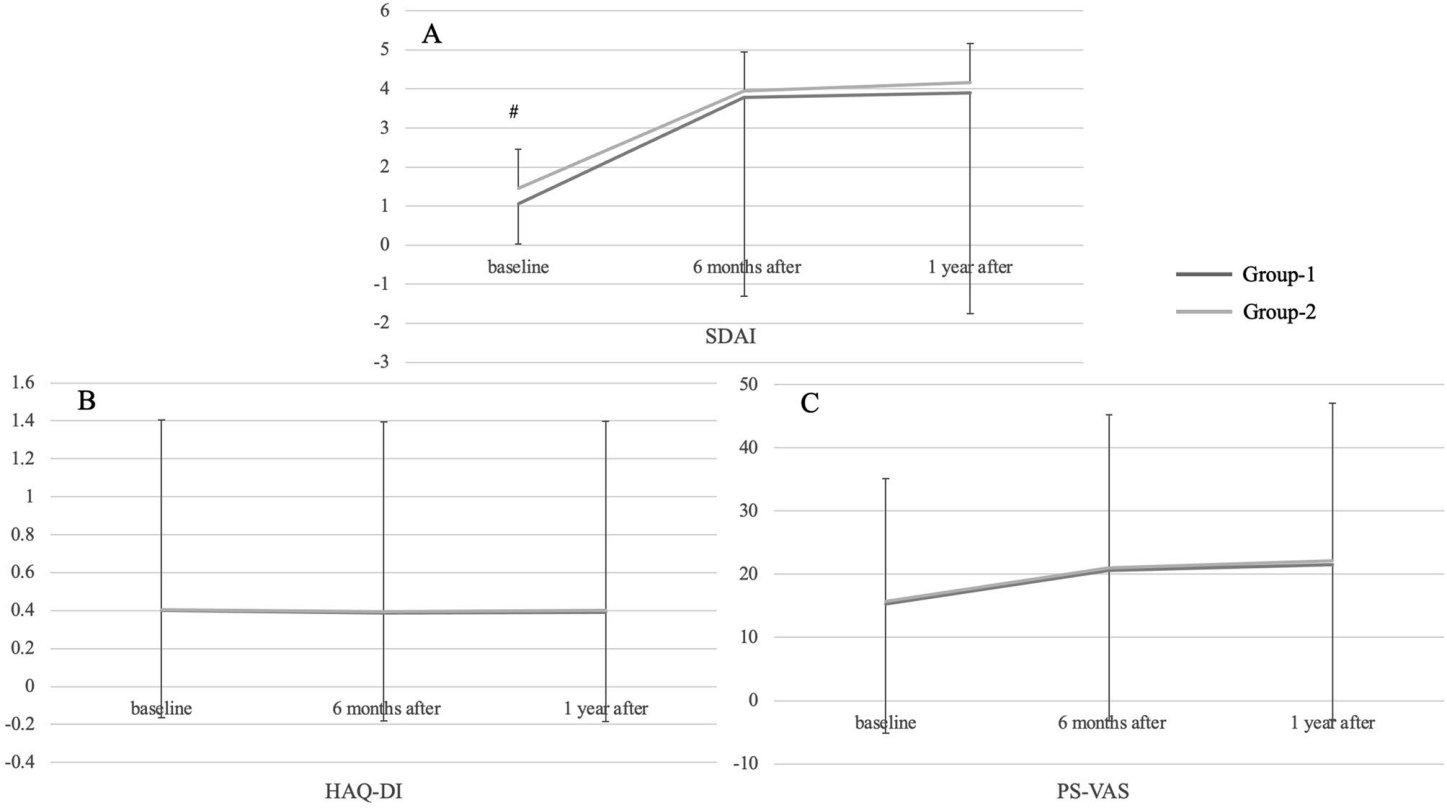
Mean SDAI score, HAQ score, and PS-VAS of the Group-1 and the Group-2 in the way one classification at baseline, 6 months after, and 1 year after each Boolean remission. (**A**) Mean SDAI of the Group-1 at baseline was significantly lower than that of the Group-2 (#; *p* < 0.0001). (**B**) Mean HAQ score demonstrated no significant difference between the two groups. C: Mean PS-VAS demonstrated no significant difference between the two groups.

Mean simplified disease activity index (SDAI) scores in Group-1 for Boolean-1 and in Group-2 for Boolean-2 were 1.07 and 1.46, 3.78 and 3.94, and 3.89 and 4.16 at baseline, 6 months after, and 1 year after, respectively. Mean SDAI score at baseline in Group-1 was significantly smaller than in Group-2. Mean Health Assessment Questionnaire Disability Index (HAQ) scores in Group-1 for Boolean-1 and in Group-2 for Boolean-2 were 0.400 and 0.403, 0.388 and 0.396, and 0.390 and 0.400 at baseline, 6 months after, and 1 year after, respectively. Mean pain score using visual analog scale (PS-VAS) in Group-1 for Boolean-1 and in Group-2 for Boolean-2 were 15.3 and 15.7, 20.6 and 21.1, and 21.6 and 22.2, at baseline, 6 months after, and 1 year after, respectively. There were no significant differences between the two groups in either HAQ score or PS-VAS (Fig. 1).

**Figure 2:**
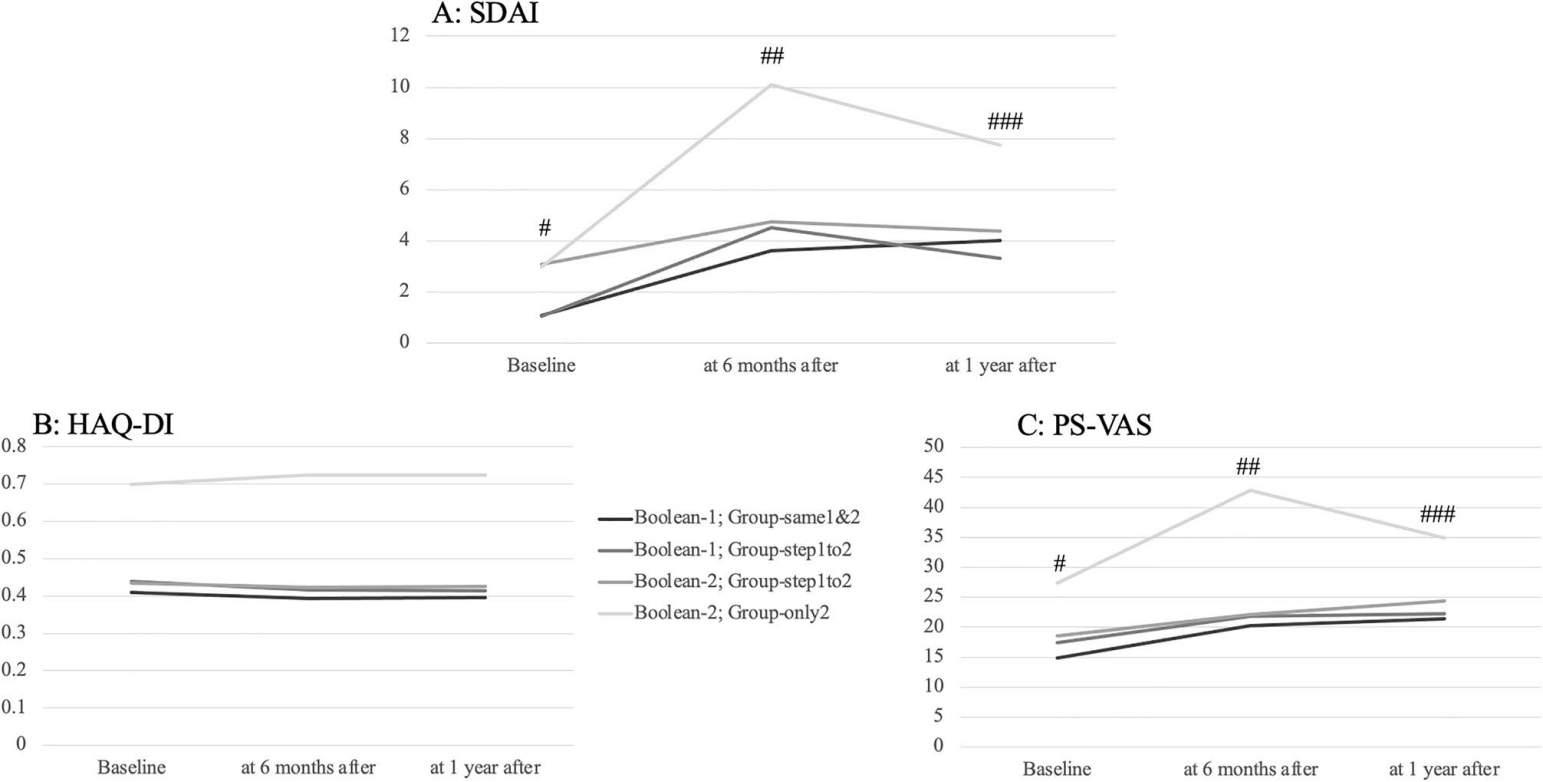
Mean SDAI score, HAQ score, and PS-VAS of the Group-same1&2, Group-step1to2, and Group-only2 in the way two classification at baseline, 6 months after, and 1 year after each Boolean remission. (**A**) Mean SDAI score of the Group-samePGA1 of Boolean-1 was significantly lower than that of the Group-step1to2 and the Group-only2 of Boolean-2 at baseline (#; *p* < 0.0001), 6 months after (##; *p* < 0.001), and 1 year after (###; *p* < 0.05), whereas mean SDAI score of the Group-step1to2 was significantly lower than that of the Group-only2 (##; *p* < 0.05). Mean SDAI score in the Group-only2 was significantly lower than that at 6 months and 12 months (*p* < 0.0001). (**B**) Mean HAQ score demonstrated no significant difference between any pairs of the groups. (**C**) Mean PS-VAS of the Group-same1&2 was significantly lower than that of the Group-step1to2 at baseline (#; *p* < 0.0001) of Boolean-2, and was lower than that of the Group-only2 at baseline (#; *p* < 0.01), 6 months after (##; *p* < 0.01), and 1 year after (###; *p* < 0.05), whereas mean PS-VAS of the Group-step1to2 was significantly lower than that of the Group-only2 at 6 months after (##; *p* < 0.05). demonstrated no significant difference between the two groups.

Mean SDAI scores in Group-same1&2 and Group-step1to2 were 1.07 and 1.06, 3.62 and 4.51, and 4.02 and 3.31 at baseline, 6 months after, and 1 year after Boolean-1, respectively. Mean SDAI score at 6 months after in Group-same1&2 was significantly smaller than in Group-step1to2. Mean SDAI scores in Group-same1&2, Group-step1to2, and Group-only2 were 1.07, 3.06, and 2.97, 3.62, 4.73, and 10.08, and 4.02, 4.39, and 7.75 at baseline, 6 months after, and 1 year after the Boolean-2, respectively. Mean SDAI score in Group-same1&2 was significantly lower than in Group-step1to2 and Group-omly2 at any time point, but the mean SDAI score in Group-step1to2 at 6 months after was significantly lower than in Group-only2 (Fig. 2A). Although the mean SDAI score at baseline was significantly lower in the Group-2 than at 6 and 12 months (*p* < 0.0001), the SDAI scores of the other groups at 6 and 12 months did not show a significant increase from baseline, there was a common trend in all groups that the mean SDAI score increased from baseline to the following period.

Mean HAQ scores in Group-same1&2 and Group-step1to2 were 0.410 and 0.440, 0.393 and 0.417, and 0.396 and 0.413, at baseline, 6 months after, and 1 year after Boolean-1, respectively. There was no significant difference between the two groups. Mean HAQ scores in Group-same1&2, Group-step1to2, and Group-only2 were 0.410, 0.435, and 0.700, 0.393, 0.423, and 0.725, and 0.396, 0.425, and 0.725 at baseline, 6 months after, and 1 year after the Boolean-2, respectively. Mean HAQ scores of Group-only2 were relatively greater compared to that of Group-same1&2 and the Group-step1to2, however, no the difference was not significant (Fig. 2B).

Mean PS-VASs in Group-same1&2 and Group-step1to2 were 14.9 and 17.5, 20.3 and 21.8, and 21.4 and 22.3 at baseline, 6 months after, and 1 year after the Boolean-1, respectively. There was no significant difference between the two groups. Mean PS-VASs in Group-same1&2, Grpup-step1PGA, and Group-only2 were 14.9, 18.6, and 27.4, 20.3, 22.1, and 42.9 at baseline, 6 months after, and 1 year after the Boolean-2, respectively. Mean PS-VAS of Group-same1&2 were significantly lower than that of Group-step1to2 at baseline of the Boolean-2, whereas mean PS-VAS of Group-step1to2 was significantly lower than that of Group-only2 at 6 months after the Boolean-2. PS-VASs of Group-same1&2 were significantly lower than that of Group-2 (Fig. 2C).

## Discussion

Our institute is the only RA center in the region (population approximately 100,000), covering 80% of RA patients. More than 90% of patients in the region have completed treatment, and approximately 70% of patients with RA are receiving treatment. Although the population is small, it is almost complete as a medical area, and it seems to be appropriate as a material for the regional cohort study.

The results of the present study suggest that both PGA ≤ 2 and PGA ≤ 1 can ensure a stable clinical course and maintenance of ADL after achieving Boolean remission. The way one results showed no statistically significant differences in PGA ≤ 1 and PGA ≤ 2, except for SDAI at Boolean remission acquisition. However, this is because most patients achieved both Boolean-1 and Boolean-2 at the same time. After achieving PGA ≤ 2, the SDAI score in the Group-step1to2 was significantly inferior to that of the Group-same1&2. There was no significant difference, however in the Group-2, the HAQ score tended to be high all the time. PS-VAS also tend to making high scores.

HAQ scores and SHS did not change significantly after baseline in all groups. These parameters are important for assessing whether clinical remission leads to global remission, but these indicators are not as sensitive as disease activity, and these parameters require more time to show changes. There was no significant difference between the groups in the present study.

These results suggest that RA patients may be divided into two groups: one group in which clinical symptoms resolve abruptly by therapeutic intervention and the other group in which clinical symptoms gradually improve. Incorporating a PGA ≤ 2 into the remission criteria appears to be at greater risk of misjudging clinical remission in a group with gradual improvement. Patients in the current study also tend to have higher disease activity after achieving Boolean remission, but it is also feared that incorporating a PGA ≤ 2 into the remission criteria increases the risk of further increased disease activity after achieving remission. Adopting PGA ≤ 2 in the Boolean remission criteria has a determinant risk.

We adopted SDAI in order to compare the two Boolean remission criterion, because we need to include evaluator’s global assessment (EGA) in the criteria. EGA has an comprehensive evaluation that includes objective viewpoint. Therefore, SDAI would be a sensitive index for the comparison between the two. In all groups, SDAI score tend to have increased after the first Boolean remission, probably because SDAI responds to mild pain and elevated CRP during maintenance therapy. In the Group-same1&2, there was no significant difference between acquisition and subsequent SDAI. The same applies to the Group-step1to2. However, there was a significant increase in the Group-2. This also indicates the risk of adopting PGA ≤ 2.

Therefore, it is concluded that the two groups clearly have a course of exacerbation, and the Boolean remission criterion using PGA ≦ 1 is superior to that using PGA ≦ 2 in terms of preventing relapse of RA disease activity.

There are some limitations in the research. One is a single-center study that has the risk of collecting maldistributed patients, but the institute is one center for rheumatic diseases in the region. Second, the number of patients with Group-2 was small to make a statistically significant difference. Previous study have reported that a part of patients tend to tick PGA between 1 and 2^11^. It is undeniable that there is a bias towards Group-2 in the current study. Third, because this study was observed up to 1 year after baseline, there are no data on long-term structural changes. That is the weakest point of this study. However, the HAQ scores of Boolean-2 of Group-step1to2 and Group-only2 are clearly different. Because activities of daily living are more important than structural changes, these results may be sufficient to clarify the risk of adopting Boolean-2 as a criterion for remission. One more risk in the Group-only2 is curious background; relatively higher age, lower ACPA and RF positive rate, higher HAQ score, and higher SHS. These made a conjecture that the Group-only2 included other comorbidities such as osteoarthritis or crustal associated arthritis, and that majority of the Group-only2 was elderly onset RA. However, the use of PGA ≤ 2 rather than PGA ≤ 1 in the Boolean remission criteria shares an important message in this study that it shares the risk of including patients inappropriate for determining remission with a stable clinical course after acquisition.

## Materials and methods

Patients who met the ACR/EULAR classification criteria for RA^14^ and were followed continuously without serious infections nor acute incidental events such as stroke, cardiovascular event, and others, were recruited. Our treatment protocol for RA is based on a T2T strategy that aims for clinical remission with simplified disease activity index (SDAI) within 6 months^15^. Patients were interviewed, and were measured components of SDAI, HAQ, and PS-VAS at least every 3 months.

Boolean remission criteria was set in two levels; Boolean-1, all of four components (TJC, SJC, CRP, and PGA) achieved ≤ 1; Boolean-2, TJC, SJC, and CRP achieved ≤ 1, but PGA ≤ 2. Recruited patients were furtherly selected in the analysis of whom disease activity matched Boolean-1 or Boolean-2 once or more in treating, and they were followed up at least one year after acquisition.

Patients were classified into groups using two ways of classification according to the first acquisition of Boolean remission criteria. The way one; Group-1, a patient group who attained Boolean-1; Group-2, a patient group who attained Boolean-2. The way two; Group-same1&2, a patient group who attained Boolean-1 and Boolean-2 at the same time; Group-step1to2, a patient group who attained Boolean-2 first and then attained Boolean-1 later; Group-only2, a patient group who attained Boolean-2 but not achieved Boolean-1. We set the date when achieved each of Boolean remissions as baseline. Mean SDAI score, HAQ score, and PS-VAS for each group in each of the two ways were calculated, and compared their mean values using Mann–Whitney U-test. Mean values of each parameter at 6 months and 1 year after the baseline were also compared using Mann–Whitney U-test as well. Mean SDAI score at baseline and subsequent period for each group was compared statistically using paired T-test.

### Software used in the statistical procedures

All the statistical procedures were performed using StatPlus:mac® (AnalystSoft, Inc., Walnut, CA, USA), and significance level was set within 5%.

### Ethics and consent

The study protocols and patient consent requirements were approved by Yoshii Hospital Ethics Committee (approval number: Y-2020-RA-2). The subjects and their families were informed that the personal information obtained in this study was anonymous and would only be used for analysis. Informed consent was obtained from all participants enrolled in the study and all subjects and their families provided signed consent.

## Methods confirmation statement

All of methods in this study were carried out in accordance with relevant guidelines and regulations.

## Data Availability

The datasets used and/or analyzed during the current study are available from the corresponding author on reasonable request.

## Abbreviations

T2T: Treat-to-target
RA: Rheumatoid arthritis
ACR: American College of Rheumatology
EULAR: European League against Rheumatism
TJC: Tenderness joint count
SJC: Swollen joint count
PGA: Patient’s global assessment
CRP: C-reactive protein
ADL: Activities of daily living Boolean-1, Boolean remission with PGA ≤ 1, TJC ≤ 1, SJC ≤ 1, and CRP ≤ 1
Boolean-2: Boolean remission with PGA ≤ 2, TJC ≤ 1, SJC ≤ 1, and CRP ≤ 1
Group-1: A patient group who attained Boolean-1 remission
Group-2: A patient group who attained Boolean-2
Group-same1&2: A patient group in the Group-1 who attained Boolean-1 and Boolean-2 remissions at the same time
Group-step1 to 2: A patient group in the Group-1 who attained Boolean-2 remission first and then attained Boolean-1 remission
Group-only2: A patient group who attained Boolean-2 remission but not attained Boolean-1 remission
SDAI: Simplified disease activity index
PS-VAS: Pain score using visual analog scale
HAQ: Health Assessment Questionnaire Disability Index

## References

[1] Smolen JS (2010). for the T2T Expert Committee: Treating rheumatoid arthritis to target: recommendations of an international task force. Ann Rheum Dis.

[2] Burmester GR, Pope JE (2017). Novel treatment strategies in rheumatoid arthritis. Lancet.

[3] Aletaha D, Wang X, Zhong S, Florentinus S, Monastiriakos K, Smolen JS (2020). Differences in disease activity measures in patients with rheumatoid arthritis who achieved DAS, SDAI, or CDAI remission but not Boolean remission. Semin. Arthritis Rheumat..

[4] Felson DT (2011). American College of Rheumatology/European League Against Rheumatism provisional definition of remission in rheumatoid arthritis for clinical trials. Arthritis Rheum.

[5] Felson DT (2011). American College of Rheumatology/European League against Rheumatism provisional definition of remission in rheumatoid arthritis for clinical trials. Ann Rheum Dis.

[6] Baker KF (2019). Predicting drug-free remission in rheumatoid arthritis: A prospective interventional cohort study. J. Autoimmun..

[7] Fusama M (2015). Psychological state is related to the remission of the Boolean-based definition of patient global assessment in patients with rheumatoid arthritis. Mod. Rheumatol..

[8] Xie W, Li G, Huang H, Zhang Z (2021). Boolean low disease activity in rheumatoid arthritis: Experience from a large real-world cohort. Rheumatol Ther.

[9] Hirsh J (2019). Limited health literacy and patient confusion about rheumatoid arthritis patient global assessments and model disease states. Arthritis. Care Res. (Hoboken).

[10] Studenic P (2020). Testing different thresholds for patient global assessment in defining remission for rheumatoid arthritis: Are the current ACR/EULAR Boolean criteria optimal?. Ann. Rheum. Dis..

[11] Ferreira RJO, Duarte C, Ndosi M, de Wit M, Gossec L, da Silva JAP (2018). Suppressing inflammation in rheumatoid arthritis: does patient global assessment blur the target? A practice-based call for a paradigm change. Arthritis Care Res. (Hoboken).

[12] Furu M (2014). Discordance and accordance between patient's and physician's assessments in rheumatoid arthritis. Scand. J. Rheumatol..

[13] Studenic P, Smolen JS, Aletaha D (2012). Near misses of ACR/EULAR criteria for remission: effects of patient global assessment in Boolean and index-based definitions. Ann. Rheum. Dis..

[14] Aletaha D (2010). 2010 Rheumatoid arthritis classification criteria: an American College of Rheumatology/European League Against Rheumatism collaborative initiative. Arthritis. Rheumatol..

[15] Smolen JS (2020). EULAR recommendations for the management of rheumatoid arthritis with synthetic and biological disease-modifying antirheumatic drugs: 2019 update. Ann. Rheum. Dis..

